# Temporomandibular Disorders among Dental Students in Pakistan: Assessment of Prevalence, Severity, and Associated Factors Based on Questionnaire

**DOI:** 10.1155/2023/8895544

**Published:** 2023-07-18

**Authors:** Muhammad Ashraf Nazir, Faisal Izhar, Shafia Hassan, Maha Tanvir, Faris Nemat, Muhammad Waleed Ashraf, Abdulaziz Alamri

**Affiliations:** ^1^Department of Preventive Dental Sciences, College of Dentistry, Imam Abdulrahman Bin Faisal University, P O Box 1982, Dammam 31441, Saudi Arabia; ^2^Department of Community & Preventive Dentistry, FMH College of Medicine & Dentistry, Lahore, Pakistan; ^3^University of Toronto, Faculty of Dentistry, 124 Edward Street, Toronto, Ontario M5G 1G6, Canada; ^4^University of Western Ontario, Faculty of Health Science, 1151 Richmond St, London, Ontario N6A 5B9, Canada

## Abstract

**Objective:**

To evaluate the prevalence, severity, and associated factors of temporomandibular disorder (TMD) among dental students.

**Methods:**

This cross-sectional study was performed on undergraduate dental students from four dental colleges in Punjab, Pakistan. Fonseca's questionnaire was used to measure the prevalence and severity of the TMD among the study participants. Bivariate and multiple logistic regression analyses were performed.

**Results:**

Of 364 dental students, 323 returned the completed questionnaires and the response rate of the study was 88.7%. The study included 52.6% males and 47.4% females. The prevalence of TMD was 66.9% with mild TMD in 40.90%, moderate TMD in 14.6%, and severe TMD in 11.50% of the participants. Psychological stress (29.6%), malocclusion (20%), and hypersensitivity (19.5%) were common among participants. The mean TMD score of the sample was 31.54 ± 24.86 which was significantly higher among participants with no/school-educated mothers (*P*=0.021) and fathers (*P*=0.002). The participants with arthritis (72.81 ± 32.19) and malocclusion (59.46 ± 31.09) and those who received orthodontic treatment (53.21 ± 34.21) demonstrated higher TMD. After controlling for other study variables, the participants with arthritis were 4.71 times more likely to have moderate/severe TMD (*P*=0.042) than those without arthritis. Similarly, the participants with malocclusion had significantly higher odds (OR = 3.57, *P*=0.029) of having moderate/severe TMD than those without malocclusion.

**Conclusion:**

This sample of dental students demonstrated a high prevalence and severity of TMD. The participants with arthritis and malocclusion demonstrated higher TMD. The study findings underscore the importance of prevention, early diagnosis, and management of TMD among the dental students.

## 1. Introduction

According to the American Society of Temporomandibular Joint Surgeons, “temporomandibular disorder (TMD) is a collective term embracing all the problems regarding the temporomandibular joint and related musculoskeletal structures” [1]. TMD is a multifactorial condition, and several factors are involved in the initiation, progression, and aggravation of the symptoms of TMD. Clicking, sound in temporomandibular joint (TMJ), is the most common symptom of TMD, followed by headache. The patients with TMD may demonstrate myofascial pain, morphological changes in the condyle, disc displacement, or disc derangement [2]. The individuals with parafunctional habits and bruxism are up to 4.8 times more likely to develop TMD [3]. It was reported that female patients demonstrated TMJ pain at rest, clicking of the temporomandibular joint, grinding of teeth, and pain in the masseter muscle more frequently than male patients [3, 4].

Globally, the prevalence estimates vary considerably among different populations. A review of 21 studies showed that the prevalence of TMD ranged from 2.6% to 11.4% [5]. In Jordan, the prevalence of TMD was 68.6% among university students [6]. TMD pain was reported by 25.9% female and 11.4% male Finnish university students [7]. A recent study showed that 49.7% of the university students complained of at least one sign or symptom of TMD in Saudi Arabia [8]. In Brazil, the prevalence of TMD was 71.9% in university students and TMD was significantly associated with perceived stress, mental disorders, and parafunctional habits [9]. Among dental students, previous Brazilian studies reported TMD in 53.21%–58.9% of the participants using the Fonseca questionnaire [10, 11]. Moreover, a relevant previous study from India showed that 42% of the dental students had some form of TMD [12]. In Pakistan, 42.6%–62% of the undergraduate medical and dental students presented with TMD [13, 14].

Epidemiological studies of TMD are important for assessing the prevalence and severity of the condition and associated factors to help develop evidence based preventive policies and programs. TMD among the dental students has been discussed in the literature before; nevertheless, there is a lack of data about the prevalence of TMD among the dental students in Pakistan. Additionally, the associations of gender, age, and dental and medical conditions with TMD have not been thoroughly investigated among the dental students. Therefore, this study aims to evaluate the prevalence, severity, and associated factors of TMD among the dental students in Punjab, Pakistan.

## 2. Materials and Methods

### 2.1. Study Design

This cross-sectional study was designed to evaluate the prevalence and severity of TMD among dental students using Fonseca's Anamnestic Index questionnaire. The study (FMH-03-2021-IRB-884-M) was approved by the Institutional Review Board at the FMH College of Medicine & Dentistry, Lahore, Pakistan.

### 2.2. Subjects

A calculated sample of 364 students was used in the study, and the sample calculations involved the assumptions of ±5% precision, 95% confidence level, and population size (*N* ≈ 4000). The study used a convenience sample of consenting students from private and public dental colleges. The participants were recruited from four dental colleges (de'Montmorency College of Dentistry Lahore, FMH College of Medicine & Dentistry Lahore, Lahore Medical Dental College, and University Medical Dental College, Faisalabad) of Punjab, Pakistan. The undergraduate dental program consists of four years in Pakistan. Therefore, students from all four years were included in the study. The participants with major systemic disease and/or those undergoing treatment for TMD were excluded from the study.

### 2.3. Instrument

Data were gathered using a self-administered questionnaire that included sociodemographic information, medical and dental history, and Fonseca's questionnaire. Sociodemographic information included age, gender, academic performance in the previous year, parental educational levels, and monthly family income. The medical history included arthritis, psychological stress, trauma to head, musculoskeletal disease, sleep disorder, and others conditions. The dental history included malocclusion, hypersensitivity, bruxism (grinding of teeth), bad breath (oral malodor), bleeding gums, difficulty in biting, and pain in teeth or gums. The types of dental treatment received were orthodontic treatment, crown and bridge, dentures, fillings, root canal treatment, extraction, and scaling. Fonseca's questionnaire with an anamnestic index was developed by Fonseca and is a valid and reliable instrument for measuring TMD [15]. The literature shows that the Fonseca instrument has been used in a wide variety of studies to collect TMD related information from different populations and has characteristics of multidimensional evaluation [10, 16, 17]. The questionnaire consists of 10 items that inquire regarding difficulty in opening mouth, problem in moving jaw to sides, muscle pain or fatigue on chewing, feeling headache, having neck pain and ear pain, noise from the temporomandibular joint on chewing or opening the mouth, grinding of teeth, problems with biting teeth together, and feeling nervous. Each item of the questionnaire is answered with “yes,” “no,” and “sometimes” options, and only one option is marked by the study participants [15]. The questionnaire was pilot tested among 30 dental students to make sure that there were no problems with readability and understanding of the questionnaire by the participants.

### 2.4. Procedure

Students were briefed about the study (purpose, details, and benefits of the study), voluntary participation of study participants, and confidentiality and privacy of their responses. They received instructions about the questionnaire and were provided with explanations if they had difficulty understanding the questions. Students who were willing to participate in the study received hard copies of the self-administered questionnaire in their classes and, after completion, they returned the questionnaires to the researchers.

### 2.5. Statistical Analysis

Statistical Package for Social Sciences Version 22.0 (IBM SPSS Statistics for Windows, Version 22.0. Armonk, NY: IBM Corp) was used for statistical analysis. Frequency distributions, means, and standard deviations were calculated for the study variables. The total score of the Fonseca questionnaire was calculated by summing up the responses of each item. A score of 10 was given to “Yes”; a score of 5 for “Sometimes”; and 0 for “No” options. The total score of the Fonseca questionnaire ranges from 0 to 100 which was used to evaluate the severity of TMD according to Fonseca index classification (Table 1).

The mean score of the Fonseca questionnaire was compared with gender, academic year, academic score, parental education, and family income using an independent samples *t*-test. Bivariate and multiple logistic regression analyses were performed to evaluate the association of independent variables (gender, academic year, academic score, parental education and family income, and medical and dental history) with the dependent variable (moderate/severe TMD). The undergraduate dental program in Pakistan consists of four years with the first and second years being preclinical years and the third and fourth years being clinical years. An academic score equal to or above 80% was categorized as a high score and below 80% as a low score. The statistical significance level was set at 5%.

## 3. Results

Of 364 dental students, 323 returned the completed questionnaires and the response rate of the study was 88.7%. The study included 64.1% of the participants from private and 35.9% from public colleges. There were 52.6% males and 47.4% females, and most participants had a high monthly family income (75.4%), and their fathers (80.8%) and mothers (75.5%) had college/university education. The mean TMD score of the sample was 31.54 ± 24.86. A significantly higher mean TMD score was observed among participants whose fathers (*P*=0.002) and mothers (*P*=0.021) had no/school education compared with those who had college/university educated parents. The study demonstrated no statistically significant differences in TMD score between males and females (*P*=0.453), participants from public and private colleges (*P*=0.627), participants from preclinical and clinical years (*P*=0.174), participants with low and high academic scores (*P*=0.733), and those with low and middle/high monthly family income (*P*=0.098) (Table 2).

Table 3 presents data on TMD and its relationship with medical and dental history, as well as dental treatment. Psychological stress (29.6%), malocclusion (20%), and hypersensitivity (19.5%) were common among participants. Regarding medical history, the participants with arthritis showed the highest mean TMD score (72.81 ± 32.19), followed by trauma to the head (41.0 ± 27.67) and musculoskeletal disease (40.50 ± 29.29). Regarding dental history, the mean TMD score was the highest among participants with malocclusion (59.46 ± 31.09) and the lowest with bleeding gums (24.46 ± 17.11). Regarding dental treatment, the participants who received orthodontic treatment showed the highest TMD severity (53.21 ± 34.21), whereas the participants with scaling demonstrated the lowest mean TMD score (24.60 ± 17.83).

**Figure 1:**
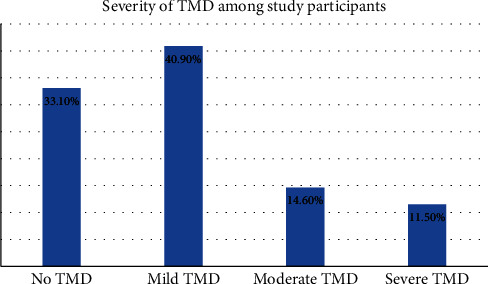
Distribution of categories of TMD among the study participants.

Figure 1 shows the distribution of study participants with different levels of TMD. Only 33.10% of the participants reported no TMD, whereas 66.9% of the participants demonstrated TMD with mild in 40.90%, moderate in 14.6%, and severe in 11.50%.

The participants with no/school-educated fathers had significantly higher odds (OR = 2.76, *P* < 0.001) of developing moderate/severe TMD than those with college/university educated fathers. Similarly, participants with no/school-educated mothers were significantly more likely (OR = 2.33, *P*=0.002) to have moderate/severe TMD than those with college/university educated mothers. Statistically higher odds of moderate/severe TMD were found among participants with arthritis (OR = 14.4, *P* < 0.001), malocclusion (OR = 6.06, *P* < 0.001), and those who received orthodontic treatment (OR = 3.08, *P* 0.001) (Table 4).

Statistically significant or marginally significant factors were included in the final model. Arthritis and malocclusion were significantly associated with moderate/severe TMD after controlling for parental education, monthly family income, and orthodontic treatment. The participants with arthritis were 4.71 times more likely to have moderate/severe TMD (*P*=0.042) than those without arthritis. Similarly, the participants with malocclusion had significantly higher odds (OR = 3.57, *P*=0.029) of having moderate/severe TMD than those without malocclusion (Table 5).

## 4. Discussion

The present study was conducted to evaluate the distribution and severity of TMD among dental students in Pakistan. The study showed the presence of TMD in 66.9% of our sample with 40.9% mild, 14.6% moderate, and 11.5% severe TMD. This finding is supported by the results of a recent study by Khan and Zaigham who used the Fonseca questionnaire and observed TMD in 62% of the medical and dental students in Pakistan [14]. Based on the same questionnaire, Nomura et al. reported that the prevalence of TMD was 53.21% among the Brazilian undergraduate dental students with 35.78% mild, 11.93% moderate, and 5.5% severe TMD [10]. Another study from Brazil by Rocha et al. reported TMD in 58.9% of the dental students [11]. Similarly, a relevant previous study by Alfawzan (2020) showed TMD in 45% of the dental students in Saudi Arabia [18]. According to another study of female dental students, 62.8% of the participants were shown to demonstrate mild to severe TMD [19]. In India, an investigation of TMD among the dental students by Kumar and Harshitha showed TMD in 42% of the participants [12]. In addition, many previous studies used the Fonseca questionnaire among health care students and showed prevalence estimates of 30.6% in Nepal [20], 54.2% in Saudi Arabia [21], and 72.2% in Turkey [22]. These variations in the prevalence of TMD can be attributed to the differences in study designs, sample size and sampling methods, and the measurement instrument or diagnostic criteria used to measure TMD.

A systematic review and meta-analysis of 21 articles on the prevalence of TMD reported that 19.1% population suffered from disk displacement and 9.8% from degenerative joint disease [23]. It is known that the dental students with TMD are at a significantly increased risk of developing jaw functional limitations and oral parafunctions [24]. Based on the aforementioned present study and previous relevant studies, there was a high prevalence of TMD among dental students which emphasizes the importance of preventive measures. This is further confirmed from the findings of another study where authors observed a significantly greater distribution of TMD among the dental students (80%) than nondental students (62%) [25]. Dental students with TMD related pain diagnosis such as myalgia or arthralgia were also shown to experience significantly higher pain intensity than those without TMD pain [24]. The etiology of TMD is not fully understood, and being a multifactorial disorder, multimodal treatment approaches should be used for individuals with TMD [26]. Therefore, the stakeholders in dental academia and healthcare systems should develop policies to prevent and manage temporomandibular dysfunctions among dental students to reduce the burden of this disease including improvement in the quality of life and learning experience.

Approximately one-third of the dental students (29.6%) reported psychological stress in our sample of students. A previous study of medical and dental students in Pakistan showed a lower prevalence of stress among dental students (12.2) than our estimates. On the contrary, Abbasi et al. reported that 68% of the dental students in their clinical year suffered from high stress in Pakistan [27]. The differences in these prevalence estimates can be attributed to the differences in the measuring instrument, study sample, and geographic location. Over the years, research has shown that stress is common among dental students which is related to demanding curriculums, course and clinical requirements, examinations, and patient management and can adversely affect their academic performance, general health, and quality of life [11, 28]. The relationship between stress and TMD among dental students has been reported in the literature [11]. However, the present study did not find a significant relationship between psychological stress and TMD. This is in line with the results of a study by Lövgren et al. who found no difference in psychological factors between dental students with or without a TMD diagnosis [24].

In the presents study, 20% of the dental students had malocclusion who demonstrated the second highest score of TMD. The study also showed a statistically significant association between malocclusion and moderate/severe TMD where dental students with TMD were 3.57 times more likely to have TMD than those without malocclusion. The association between TMD and malocclusion was confirmed by Thilander et al. who found that anterior open bite, posterior crossbite, increased overjet, and Angle class III malocclusion were associated with TMD [29]. As demonstrated in the present study, dental students are at risk of developing TMD; therefore, a routine dental examination should be performed to identify malocclusion and other oral problems and referred for early investigation and intervention.

Osteoarthritis and rheumatoid arthritis are common conditions encountered in clinical practice and are associated with TMD [30, 31]. With a global prevalence of 15%, osteoarthritis is characterized by chronic degeneration of joint tissues causing pain, and it affects stress-bearing as well as other joints in the body including the temporomandibular joint (TMJ). Osteoarthritis may be localized to the TMJ or can be part of a generalized form of this condition [30]. Rheumatoid arthritis (RA) is a chronic, systemic, immune-mediated inflammatory disease that involves symmetrical joints causing swelling, pain, and functional limitation. In a nationwide study, Lin et al. examined 17,317 patients with RA and 17,317 matched controls and found that patients with RA were at 2.53 times higher risk of developing TMD than controls [31]. In the present cross-sectional study, dental students with arthritis demonstrated the highest mean TMD score and participants with arthritis had significantly higher odds (4.71) of having moderate/severe TMD than those without arthritis.

The following limitations of this study should be considered. Data collected from four dental colleges may not represent the dental student population in Pakistan; therefore, the generalizability of study findings is limited. Furthermore, a convenience sample of dental students may lead to selection bias and influence the validity of the study. Random errors can also occur in this type of research if participants do not accurately respond to each item in the questionnaire. To the best of our knowledge, this is the first study that identified significant associations of malocclusion and arthritis with TMD among dental students. However, a cross-sectional study design is limited as far as the investigation of cause and effect relationships is concerned. Additionally, the present study included self-reported data on malocclusion and arthritis and found their significant associations with TMD. Therefore, large cohort studies should be conducted to investigate clinically diagnosed TMD and associated factors including different types of malocclusion and joint diseases among dental students.

## 5. Conclusions

This study showed a high prevalence and severity of TMD among dental students. Psychological stress and malocclusion were also common among them. The participants with arthritis and malocclusion demonstrated high TMD. Moreover, arthritis and malocclusion were significantly associated with an increased likelihood of moderate/severe TMD. The study underscores the importance of prevention, early diagnosis, and management of TMD among the dental students.

## Figures and Tables

**Table 1:** Categories of TMD according to Fonseca index classification.

Fonseca index classification	
0–15 points	No TMD
20–40 points	Mild TMD
45–65 points	Moderate TMD
70–100 points	Severe TMD

**Table 2:** TMD score and its relationship with demographic factors among study participants.

Study variables	*N* (%) *N* = 323	Mean TMD score	*P* value
Gender			
Male	170 (52.6)	30.56 ± 27.05	0.453
Female	153 (47.4)	32.64 ± 22.22	
Academic year			
Preclinical years	102 (31.6)	32.82 ± 25.61	0.174
Clinical years	221 (68.4)	28.77 ± 23.03	
Academic score in the previous year			
Low	174 (53.9)	31.14 ± 23.60	0.733
High	149 (46.1)	32.09 ± 26.4	
Father's education level			
No/school education	62 (19.2)	40.32 ± 31.22	0.002
College/university education	261 (80.8)	29.46 ± 22.67	
Mother's education level			
No/school education	79 (24.5)	37.15 ± 31.41	0.021
College/university education	244 (75.5)	29.73 ± 22.11	
Monthly family income (*N* = 195)			
Low/middle	48 (24.6)	38.12 ± 32.48	0.098
High	147 (75.4)	30.77 ± 24.38	

**Table 3:** TMD score and its relationship with medical and dental history and dental treatments among the study participants.

Medical history (*N* = 266)	*N* (%)	Mean TMD score
Arthritis	16 (6.0)	72.81 ± 32.19
Psychological stress	79 (29.6)	31.83 ± 20.76
Trauma to head	10 (3.8)	41.0 ± 27.67
Musculoskeletal disease	10 (3.8)	40.50 ± 29.29
Sleep disorder	32 (12.0)	35.78 ± 26.67
Others	17 (6.4)	25.85 ± 21.11

*Dental history (N* *=* *185)*		
Malocclusion	37 (20.0)	59.46 ± 31.09
Hypersensitivity	36 (19.5)	39.72 ± 23.54
Bruxism (grinding of teeth)	9 (4.9)	26.11 ± 25.47
Bad breath (oral malodor)	13 (7.0)	34.61 ± 23.67
Bleeding gums	37 (20.0)	24.46 ± 17.11
Difficulty in biting	12 (6.5)	39.16 ± 29.53
Pain in teeth or gums	41 (22.2)	32.44 ± 22.67

*Dental treatment received (N* *=* *245)*		
Orthodontic treatment	42 (17.1)	53.21 ± 34.21
Crown and bridge	21 (8.6)	48.57 ± 22.70
Dentures	5 (2.0)	45.0 ± 39.37
Fillings	43 (17.6)	33.14 ± 23.27
Root canal treatment	13 (5.3)	35.38 ± 19.94
Extraction	27 (11)	30.0 ± 21.57
Scaling	40 (16.3)	24.60 ± 17.83
Others	54 (22.0)	25.92 ± 18.38

**Table 4:** Bivariate analysis: association of different factors with moderate/severe TMD among the study participants.

Study variables	Unadjusted odds ratio (CI 95%)	*P* value
Gender		
Male	1.05 (0.64, 1.73)	0.841
Female		
Academic year		
Preclinical years	1.04 (0.61, 1.76)	0.897
Clinical years		
Academic score in the previous year		
High	0.97 (0.59, 1.61)	0.921
Low		
Father's education level		
No/school education	2.76 (1.54, 4.94)	<0.001
College/university education		
Mother's education level		
No/school education	2.33 (1.35, 4.01)	0.002
College/university education		
Monthly family income		
Low/middle	1.95 (0.98, 3.88)	0.055
High		
Psychological stress		
Yes	1.13 (0.64, 2.01)	0.668
No		
Arthritis		
Yes	14.40 (3.99, 51.97)	<0.001
No		
Malocclusion		
Yes	6.06 (2.94, 12.48)	<0.001
No		
Orthodontic treatment		
Yes	3.08 (1.58, 6.01)	0.001
No		

**Table 5:** Multiple logistic regression analysis: association of different factors with moderate/severe TMD among the study participants.

Study variables	Adjusted odds ratio (CI 95%)	*P* value
Father's education level		
No/school education	1.81 (0.72, 4.55)	0.203
College/university education		
Mother's education level		
No/school education	1.87 (0.79, 4.40)	0.152
College/university education		
Monthly family income		
Low/middle	0.90 (0.37, 2.19)	0.814
High		
Arthritis		
Yes	4.71 (1.06, 20.97)	0.042
No		
Malocclusion		
Yes	3.57 (1.14, 11.23)	0.029
No		
Orthodontic treatment		
Yes	1.69 (0.56, 5.07)	0.350
No		

## Data Availability

The SPSS data file of this study is available from the corresponding author upon request.
